# Supplementary material for: First demonstration of in-memory computing crossbar using multi-level Cell FeFET
Source: Nat Commun. 2023 Oct 10;14:6348. doi: 10.1038/s41467-023-42110-y (PMC10564859; doi:10.1038/s41467-023-42110-y)
Supplement: Supplementary file 1 — Supplementary Information [file 41467_2023_42110_MOESM1_ESM.pdf]

Supplementary Materials

S1 Modeling of FeFET devices

The TEM micrograph of the FeFET co-integrated into the 28 nm technology node is given in Fig. S1**a** and the associated gate stack Fig. S1**b**. The $I_{ds} - V_{gs}$ transistor curves of the 2 states- the low V_{th} (LVT) and the high V_{th} is given in Fig. S1 **c**. The 1FeFET-1R structure and the layout of the segment are shown in Fig. S1**d** and **e** respectively. Fig. S1**f** shows the simulated crossbar structure with the accumulation capacitor and the ADC connected to it. Fig. S1 shows the overall structure of the FeFET and the associated connections for the proposed MAC macro.

The FeFET is modeled by separately modeling the ferroelectric capacitor (Fe-Cap) and the underlying transistor. The Fe-Cap is modeled using the Preisach model to capture the polarization (P) - Voltage (V) characteristics [18]. This macro-level model captures the electrical relationship between the applied voltage and polarization in the ferroelectric layer. An auxiliary voltage (V_{aux}) is calculated to capture the switching characteristics. V_{aux} represents the voltage to which the ferroelectric dipoles respond after relaxation. This is given using Eq. (1).

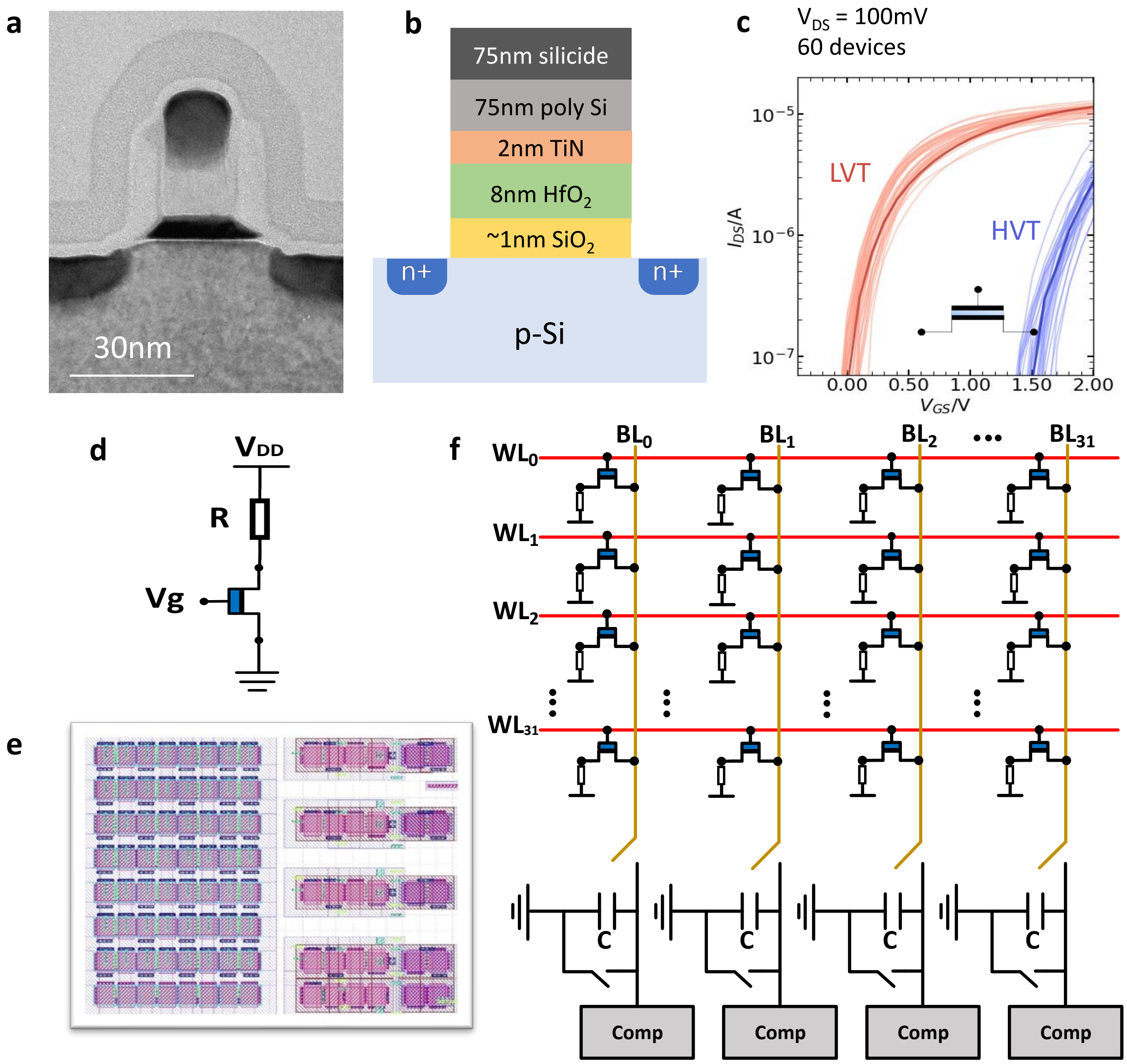

Figure S1: **Structure of the FeFET and proposed design. a.** TEM image of the FeFET integrated in 28 nm HKMG technology, **b.** material stack of the FeFETs, **c.** $I_{ds} - V_{gs}$ curves of 2 states (LVT/HVT) of 60 devices, **d.** 1FeFET-1R structure that forms the basis of our cell. **e.** Layout of the FeFET segment, and **f.** crossbar of the 32x32 cells with the accumulation capacitor.

$$V_{aux} = V_{in} - \tau_v \frac{d}{dt} V_{aux} \quad (1)$$

where τ_v represents the relaxation time for V_{aux} and V_{in} is the applied input voltage at the gate of the Fe-Cap. Then, the polarization corresponding to V_{aux} (P_{aux}) is calculated using:

$$P_{aux} = m \cdot P_s \cdot \tanh(w(V_{aux} \pm V_c)) + P_{off} \quad (2)$$

$$w = \frac{T_{fe}}{2V_c} \cdot \ln\left(\frac{P_s + P_r}{P_s - P_r}\right) \quad (3)$$

where V_c is the coercive voltage of the Ferroelectric material, P_s is the saturation polarization, P_r is the remnant polarization, m is the slope of the curve and P_{off} is the offset polarization. The upwards (-) and downwards (+) polarization determine the sign of tanh function, and the values of m and P_{off} are calculated using polarization history, which determines the P-V characteristics of the minor loops. For the main loop, $m = 1$ and $P_{off} = 0$. The calculated polarization is equated to the MOSFET gate charge and determines the region of operation. The industry-standard compact model BSIM-IMG [19] is used with calibrated parameters for the underlying MOSFET. Thus, the complete FeFET can be modelled as a Ferroelectric capacitor in series with the gate of MOSFET. Depending on the polarization in the Fe-Cap, the MOSFET is set into different V_{th}.

The shift in V_{th} of the FeFET is observed on applying different gate voltages to the

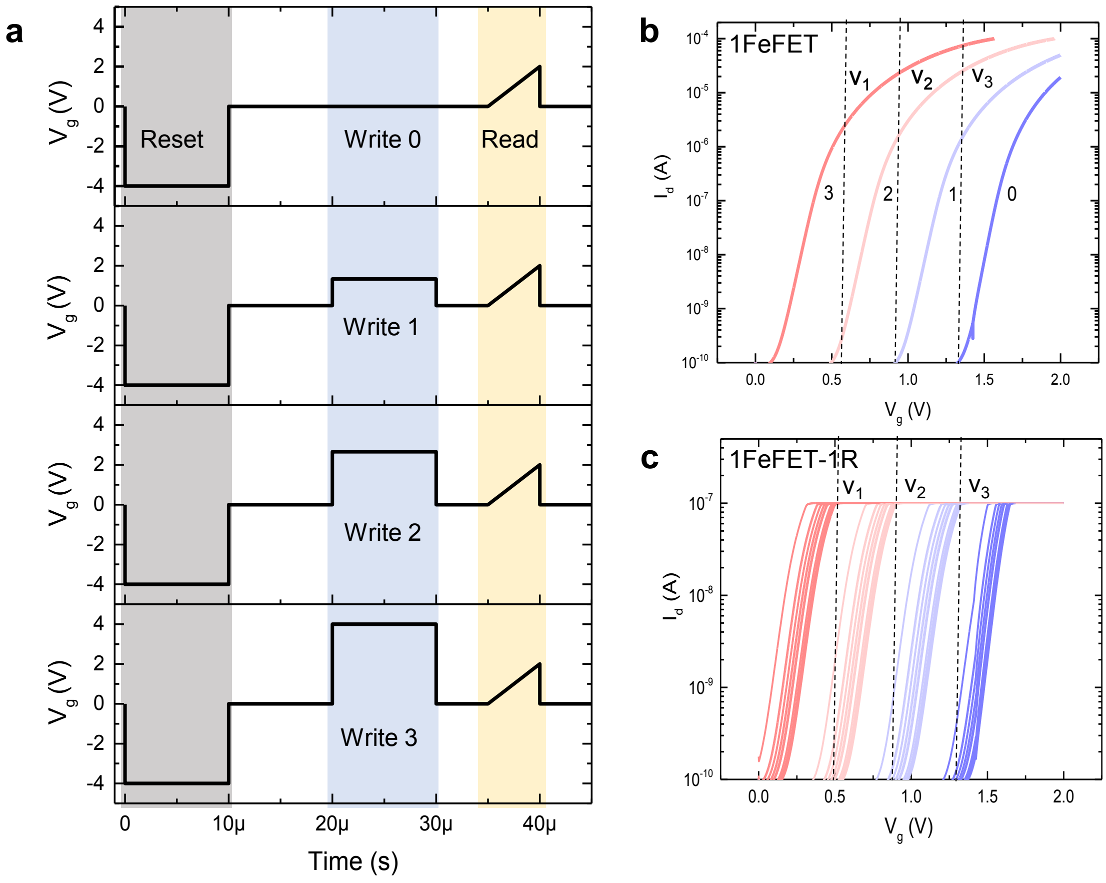

Figure S2: **Simulation results utilizing the proposed FeFET model. a.** The gate voltage applied at the FeFET to write it into 4 distinct states. Initially, a reset pulse is applied, followed by a write pulse of suitable magnitude and voltage sweep. **b.** I-V characteristics on sweeping the gate voltage (V_g) for the four different states of single FeFET during read. **c.** I-V characteristics of 1FeFET-1R for the four different states of single FeFET during read. The current is limited to 100 nA.

gate of the FeFET. Fig. S2**a** and **b** demonstrate the write and read functionality of the FeFET. The magnitude of the Write Voltage sets the different levels of polarization in the Ferroelectric layer according to Eq. (3), and the corresponding shift in V_{th} is observed in the $I_{ds} - V_{gs}$ characteristics.

To account for the variability due to the extrinsic sources of variation and the variation due to ferroelectric polarization, the V_{th} of the underlying MOSFET is varied. This can be varied using the V_{th} deviation parameter “DELVTRAND”. The measured variation of V_{th} of mean standard deviation of 40 mV is used for this work. An external resistor connected to the FeFET cell (1FeFET-1R) can drastically limit ON current variability. The corresponding $I_{ds} - V_{gs}$ curve for 100 Monte Carlo runs is shown in Fig. S2**c** As expected, due to the external Resistor ON current is constant at 100 nA despite having variability in the V_{th}. When the voltage is constant, as in our case, then integrating a high resistance reduces the current from 2 µA to 0.1 µA and as a result, a lesser power ($P = V^2/R$). As the 1FeFET-1R cell is connected to a constant supply voltage V_{dd}, the power consumed by the cell ($P = V^2/R$) is reduced.

S2 Measurement setup

For the electrical characterization a measurement setup consisting of a PXIe System from NI is used. The test-structures, consisting of 25 pads, contain up to 8 individual FeFETs. A separate NI PXIe-4143 Source Measure Unit (SMU) can access each contact of the test-structures. Source selection for each contact is handled by a custom switch-matrix that is controlled by NI PXIe-6570 Pin Parametric Measurement Units (PPMU). The external resistor is connected at the source-terminal contact on the switch-matrix. The switch-matrix connects to the FeFET-structures via a probe-card (Fig. S3 and Fig. S4).

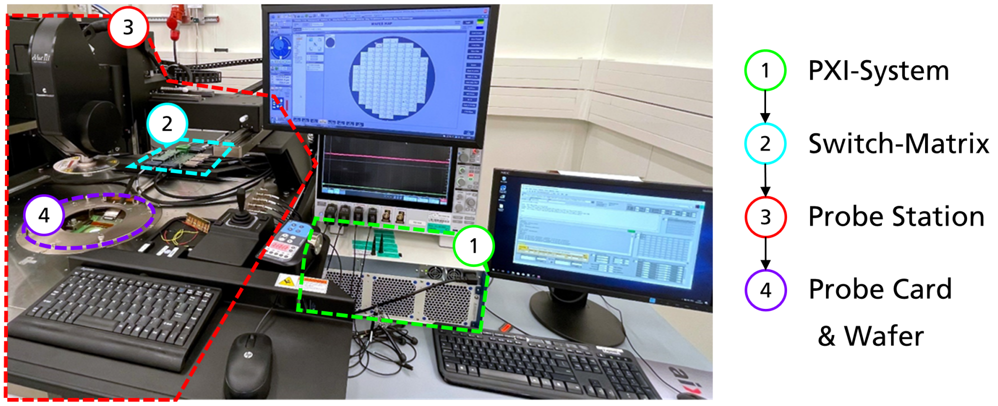

Figure S3: **Measurement setup for FeFET characterization.** A PXI System provides Source Measurement Units (SMU) and Pin Parametric Measurement Units (PPMU). PP-MUs are used to configure the Switch Matrix for routing the source signals to the respective contact needles. Test structures are available on 300 mm wafers and connected to the measurement setup on a semi-automatic probe station using a probe card.

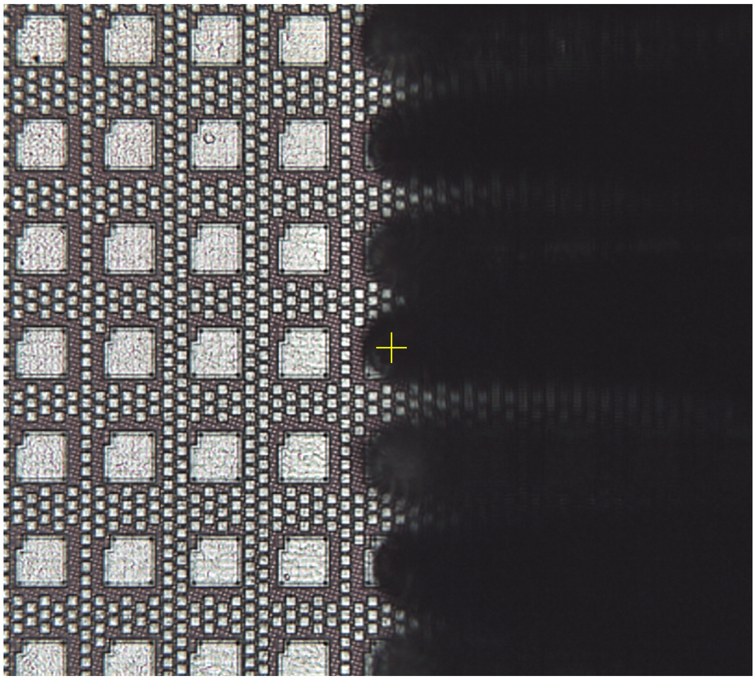

Figure S4: **Top view of the characterised test structure**. Up to 8 FeFETs with the individual source, drain and gate terminals are located in industry-standard test structures with 25 pads.

S3 Detailed measurement and writing procedure of the stored states in the FeFET

In the following, we elucidate the employed method step by step as well as the different procedures employed for measuring the V_{th} states, programming and verifying the different V_{th} states as well as measuring the retention of FeFET devices over time.

1. Structure of the fabricated FeFET-based crossbar array: The measurements are conducted on a fabricated FeFET-based crossbar array with a size of (9×7). Hence, the array consists of 9 word-lines (WL) and 7 bit-/source-lines (BL/SL). The FeFET devices are "AND" connected to form the crossbar array, as Fig. S5 illustrates. All measurements are performed at the wafer level and the temperature desired for experiments is set using a temperature-controlled chuck.

2. Procedure of V_{th} measurement: After a write voltage pulse is applied to a FeFET, the device exhibits a certain V_{th}. Reading-out the programmed/stored V_{th} state is necessary to 1) verify after writing what the exact V_{th} state is stored in the FeFET, and 2) perform later the required retention measurements, which quantifies how the stored V_{th} states may drift over time. To extract the V_{th} of FeFET, the $I_D - V_G$ transfer characteristic of FeFET needs to be first measured. To perform that for FeFETs in a certain row,

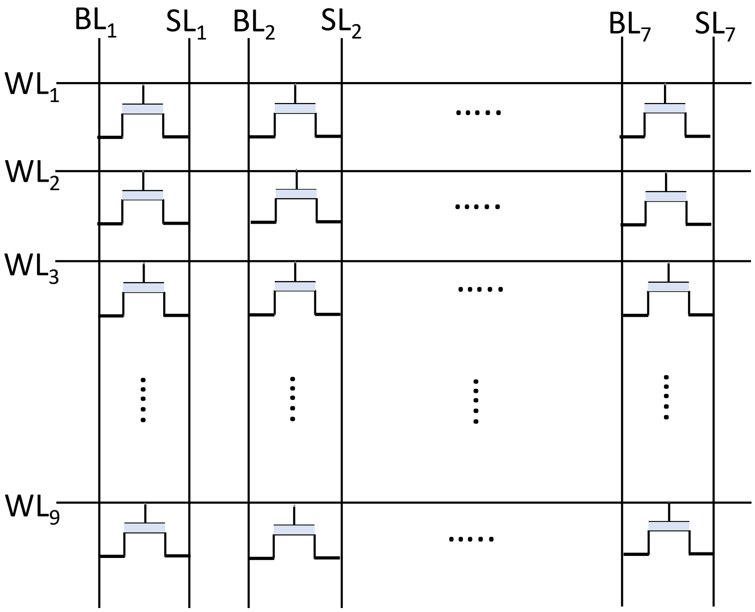

Figure S5: Structure of the AND-connected FeFET-based crossbar array (9×7) with 9 word-line (WL), 7 bit-line (BL) and 7 source-line (SL).

the corresponding WL voltage for that row is ramped from 0 V to 1.4 V with an increment of 100 mV, while applying 100 mV at the BL. The drain current I_D is then sampled at every read voltage step V_G, with a sampling time of 80 s, towards obtaining the full $I_D - V_G$ characteristic. To ensure a reliable current measurement, a settling time of 1 s is waited for every read voltage step. Finally, the V_{th} state is extracted from the measured $I_D - V_G$ curve using the standard constant-current method [?] in which the gate voltage V_G is extracted at a certain fixed I_D current of 100 nA.

3. Programming FeFET procedure: Writing FeFET devices occur at the row level in which a specific write voltage is applied to a certain WL, while all SL/BL has 0 V. To ensure programming FeFETs to a certain targeted V_{th} state, a "write-verify" scheme is

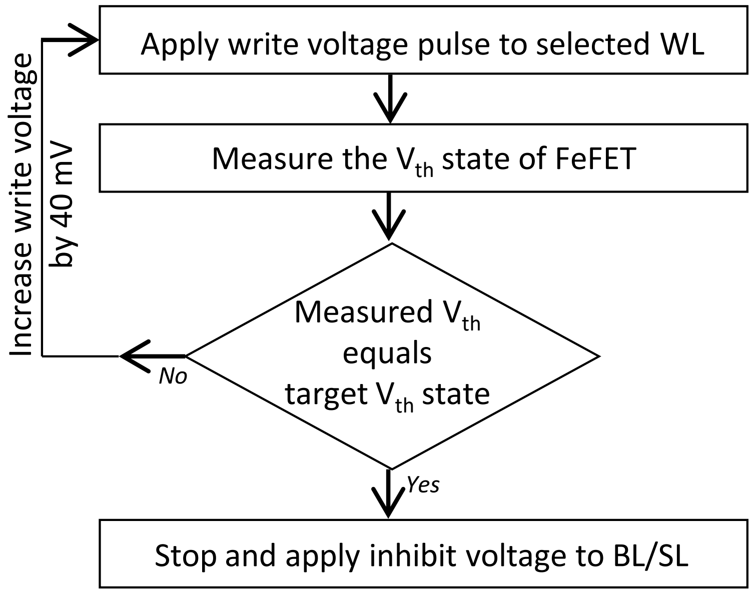

Figure S6: Schematic flowchart of the emplyoed procedure to program FeFETs into a certain target V_{th} state along with the "write-verify" scheme.

applied as follows. Fig. S6 illustrates an abstracted flowchart

First a write voltage of 2.1 V is applied for 400 ns (i.e., a write pulse of 2.1 V amplitude and 400 ns pulse width is applied). Then, a period of 2 s is waited to provide a sufficient time for any de-trapping within the FeFET. Afterwards, the V_{th} is measured to verify whether it matches the targeted level. V_{th} measurement is performed using the procedure explained in **2**. If the measured V_{th} does not match the target level, then a new write voltage pulse, with 40 mV higher amplitude, is applied. The "write-verfy" scheme is repeated until the target V_{th} is reached. It is important to note that once a specific FeFET device reaches the target V_{th}, it is changed to the "inhibit-condition" to ensure no disturbance occurs when other FeFET devices are being programmed. Inhibit is defined

as a $V_{BL} = V_{SL} = 3.2\,V$. To avoid disturbs in FeFETs sharing inhibited BL/SL along the passive WL, they are raised to $V_{WL_p} = 1.6\,V$. "Write-verify" scheme is continued until all (i.e., 7) FeFETs of the activated/selected WL reach the target V_{th} state. The previous programming procedure is then applied to the another row (i.e., WL) in order to program the FeFET devices there to another target V_{th} state.

4. Retention measurement procedure: The focus of this work is to demonstrate how 2-bit FeFET can be employed to perform in-memory computing crossbar. To realize 2-bit FeFET, four different V_{th} states should be reliably stored. Therefore, we perform retention measurements to quantify how stored V_{th} states may drift over time. To this end, four different V_{th} states (1.2 V, 0.9 V, 0.6 V and 0.3 V) are targeted to be programmed in the FeFET devices. First, the temperature desired for the experiment (85 °C) is set using the temperature-controlled chuck and then a sufficient time is waited to ensure thermal stability. Then, four rows in the crossbar array are selected and the 7 FeFET devices in each row are programmed to a certain V_{th} state (1.2 V, 0.9 V, 0.6 V, 0.3 V). The FeFET programming procedure along with the "write-verify" scheme is explained in **3**. Afterwards, the V_{th} states stored in the 7 FeFETs across one row are, in parallel, measured. The V_{th} measurement procedure is explained in **2**. The V_{th} measurements are then repeated with logarithmic time steps for 10^5 seconds, which is approximately one day. The obtain measurements provide the necessary information about the retention behaviour of FeFET devices (i.e., the drift of V_{th} over time).

S4 Device variations measurements and simulation

The achievable device variation is obtained by screening the switching transition of 60 FeFETs with an area size of 900x900 nm^2 each. The states are set by erase operation, which is typically more gradual over a wider switching range [35] due to the intrinsic current percolation path effects in MFIS-based FeFETs [36]. The erase voltages range from -2 V to -5.3 V with a pulse length of 200 ns in decrements of -50 mV. This gives us a measured variability on average of about 40 mV.

From the measured variability in V_{th}, variability in the sampling voltage is computed. 1000 Monte-Carlo runs for each stored and input state is performed. The corresponding mean, fifth percentile, and ninety-fifth percentile of the sampled voltage are shown in Fig. S7. To reduce simulation complexity, up to 4 cells in the array are simulated. For a higher number of cells, the variability is calculated algebraically. This saves the simulation time, which otherwise would have taken considerable time and computing resources.

In Eq. (2) of the main text shows that the sampling voltage is the summation of the current from all FeFETs. Therefore, the maximum variability in the sampling voltage is the summation of the variability for all the FeFETs. The sampling voltage doubles for doubling the number of cells. Therefore, for a given number of cells (n), the maximum

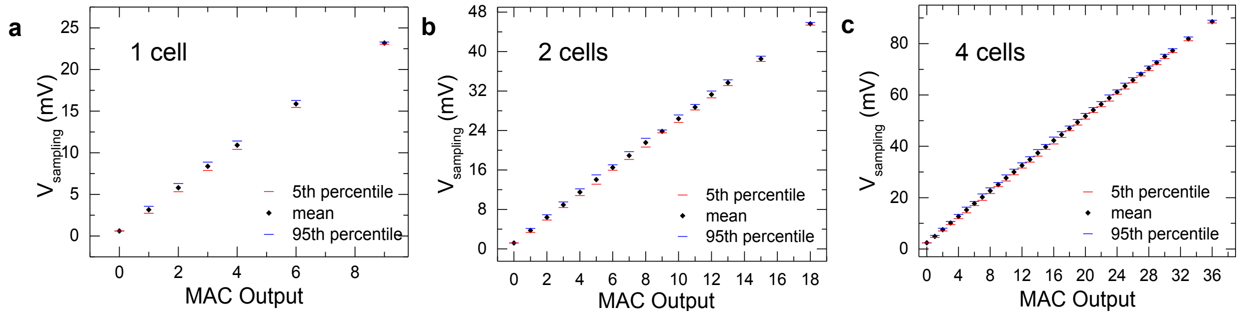

Figure S7: **Variation in MAC output due to the measured variation in V_{th}.** **a.** Mean, 5th percentile and 95th percentile of Sampling Voltage against MAC output for 1 cell, **b.** 2 cells, and **c.** 4 cells. Monte Carlo simulations of 1000 runs for each input and stored case is performed with a standard deviation of 40 mV in V_{th}.

standard deviation is given by :

$$\sigma V_{sampling,ncells} = 2 * \sigma V_{sampling,n/2cells} \tag{4}$$

The predicted sampling voltage matches very well with simulations for up to 4 cells. Thus, for predicting the variability in sampling voltage for 32 cells, results from 4 FeFET are extrapolated. The maximum σ $V_{sampling}$ is 0.16 mV for a single cell. For the case of 4 cells, the maximum σ $V_{sampling}$ from simulations is 0.44 mV. From our predictions using the equation above, the maximum sigma is 0.48 mV. This validates the equation above to calculate the variability for a higher number of cells. σ $V_{sampling}$ for 32 cells is calculated to be 3.52 mV. The final variability for 32 cells in the array is used to analyze the loss in inference accuracy for the neural network.

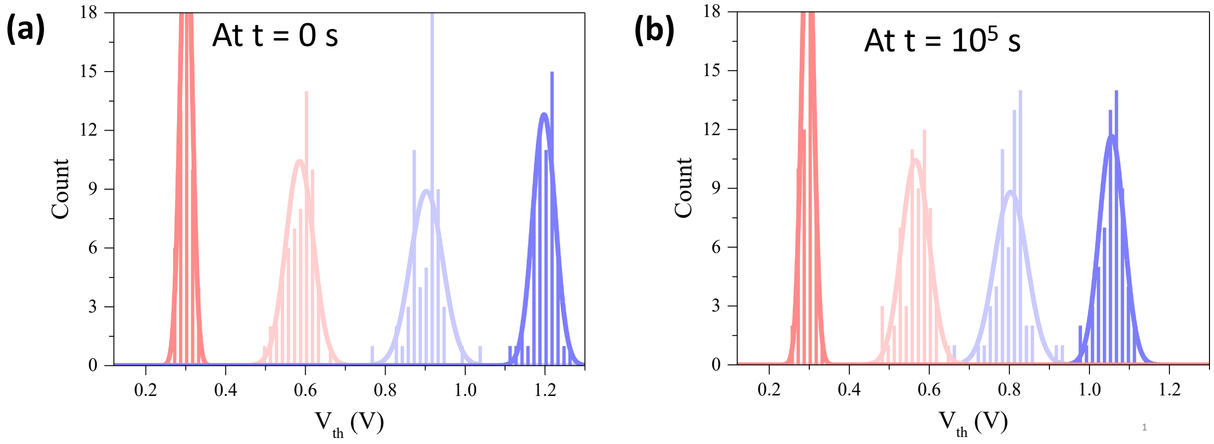

Figure S8: **Distribution of the threshold voltage states at t=0 s and at t=10 ^{5} s showing minimal overlap between the states.**

S5 Neural network implementation

Weights and activations are quantized to 2 bits with unsigned format. The initial transformation from FP to quantized values is computed by (5), where a_{FP}, a_{quant} are FP and quantized tensors respectively, b is the target bit-width and Δ_a is the scaling factor.

$$a_{quant} = \text{clip}\left(\text{round}\left[\left(\frac{a_{FP}}{\Delta_a}\right), \{-2^{b-1}, 2^{b-1}-1\}\right]\right) \tag{5}$$

The scaling factor Δ_a is obtained by:

$$\Delta_a = \frac{\max(|a_{FP}|)}{2^{b-1}-1} \tag{6}$$

To efficiently used the proposed IMC architecture, we need to enable unsigned quantization of weights w and activations x. Therefore, zero-points z_w, z_x are used as an offset to have all quantized values > 0. The resulting unsigned quantization is computed as

follows (e.g. for activations x):

$$x_{quant,unsigned} = x_{quant} + z_x = x_{quant} + (-\min(x_{quant})) \quad (7)$$

Assuming that weights and activations tensors have been transformed to matrices, The matrix multiplication between activation matrix X_{quant} of size $m \times n$ and weights matrix W_{quant} of size $n \times p$ outputs matrix Y_{quant} $(m \times p)$. The element $y_{j,k} \in Y$ is computed as follows:

$$y^{j,k}_{quant} = \sum_{i=0}^{n} x^{j,i}_{quant} w^{i,k}_{quant} - \sum_{i=0}^{n} x^{j,i}_{quant} z_w - \sum_{i=0}^{n} w^{i,k}_{quant} z_x + \sum_{i=0}^{n} z_x z_w \quad (8)$$

The output is then re-scaled to FP precision by multiplying Y_{quant} with the scaling factor $\Delta_x \times \Delta_w$. Thanks to the presence of ReLU at the output of all layers, z_x is zero for all layers except the first one that receives the input data.
